# Dead or alive: microbial viability treatment reveals both active and inactive bacterial constituents in the fish gut microbiota

**DOI:** 10.1111/jam.15113

**Published:** 2021-05-04

**Authors:** T.P.R.A. Legrand, M.L. Wos‐Oxley, J.W. Wynne, L.S. Weyrich, A.P.A. Oxley

**Affiliations:** ^1^ School of Biological Sciences The University of Adelaide Adelaide SA Australia; ^2^ CSIRO, Agriculture and Food Hobart Tas Australia; ^3^ South Australian Research and Development Institute Aquatic Sciences Centre West Beach SA Australia; ^4^ College of Science and Engineering Flinders University Adelaide SA Australia; ^5^ Department of Anthropology and Huck Institutes of Life Sciences The Pennsylvania State University University Park PA USA; ^6^ Faculty of Science Engineering and Built Environment School of Life and Environmental Sciences Deakin University Geelong Vic. Australia

**Keywords:** 16S rRNA, fish, gut, microbiota, PMA, viability, yellowtail kingfish

## Abstract

**Aims:**

This study evaluated the microbial viability of fish gut microbiota in both digesta (faecal) and mucosal samples using a modified propidium monoazide (PMA) protocol, followed by 16S ribosomal RNA (rRNA) gene sequencing.

**Methods and results:**

Digesta and gut mucosal samples from farmed yellowtail kingfish (*Seriola lalandi*) were collected and a modified PMA treatment was applied prior to DNA extraction to differentiate both active and nonviable microbial cells in the samples. All samples were then sequenced using a standard 16S rRNA approach. The digesta and mucosal samples contained significantly different bacterial communities, with a higher diversity observed in digesta samples. In addition, PMA treatment significantly reduced the microbial diversity and richness of digesta and mucosal samples and depleted bacterial constituents typically considered to be important within fish, such as Lactobacillales and Clostridales taxa.

**Conclusions:**

These findings suggest that important bacterial members may not be active in the fish gut microbiota. In particular, several beneficial lactic acid bacteria (LAB) were identified as nonviable bacterial cells, potentially influencing the functional potential of the fish microbiota.

**Significance and impacts of the study:**

Standardizing the methods for characterizing the fish microbiota are paramount in order to compare studies. In this study, we showed that both sample type and PMA treatment influence the bacterial communities found in the fish gut microbiota. Our findings also suggest that several microbes previously described in the fish gut may not be active constituents. As a result, these factors should be considered in future studies to better evaluate the active bacterial communities associated with the host.

## Introduction

Nucleic acid sequence‐based techniques have greatly improved our understanding of microbial communities living in and around animals. More specifically, these approaches have elucidated the involvement of the microbiome in the health and disease of numerous hosts, including fish (Chow *et al*. 2010; Kostic *et al*. 2013; de Bruijn *et al*. 2018; Legrand *et al*. 2020b; Wynne *et al*. 2020). In addition to supporting the host immune system functions and combatting pathogens, the fish microbiota has been shown to be involved in numerous other functions, such as nutrient metabolism, digestion, reproduction and the recycling of waste products (van Kessel *et al*. 2016; Banerjee and Ray 2017; Butt and Volkoff 2019). This wealth of information is of particular value for the fish farming industry, as such knowledge can be applied to improve fish health and performance (Legrand *et al*. 2020b).

Next generation sequencing (NGS) techniques are not without caveats, and despite efforts in developing standardized approaches (Vatsos 2017; Poussin *et al*. 2018), care needs to be taken when drawing conclusions from fish microbiome related studies. For instance, sample collection, laboratory procedures, sequencing and data analysis can widely differ between studies, limiting reproducibility and resulting in differing conclusions (Pollock *et al*. 2018; Poussin *et al*. 2018; Legrand *et al*. 2020b). Different sample types (e.g. mucosa and digesta) also produce distinct microbiota; different samples from Atlantic salmon (*Salmo salar*) (Gajardo *et al*. 2016) and rainbow trout (*Oncorhynchus mykiss*) (Lyons *et al*. 2017) have been shown to exhibit different bacterial communities. Furthermore, metagenomics techniques are often performed using genomic DNA (gDNA) from the collected samples, limiting the ability to differentiate viable and nonviable microbial cells.

Several techniques have been developed to differentiate between viable and nonviable cells when using NGS. For instance, RNA instead of gDNA can be used to generate libraries and thus better characterize the active constituents of the host gut microbiota (De Vrieze *et al*. 2018; Legrand *et al*. 2020a; Legrand *et al*. 2020c). Alternative methods that assess microbial viability of gDNA in samples can also be utilized, such as molecular viability testing (MVT) and viability PCR (vPCR) (Cangelosi and Meschke 2014). The latter is by far the most studied method, which assesses viability based on cell envelope impermeability where samples are pretreated with a membrane‐impermeative reagent such as propidium monoazide (PMA) (Nocker and Camper 2009; Cangelosi and Meschke 2014). During treatment, this reagent covalently binds to free DNA and nucleic acids in cells that do not have intact cell membranes which, following photoactivation, enables them to be separated from viable cells with intact membranes prior to DNA extraction and PCR amplification, and thus interfering with downstream sequencing (Cangelosi and Meschke 2014). Due to this advantageous property, PMA has thus been used on a wide range of samples to discriminate between viable and nonviable bacterial cells and/or spores in microbiome‐related studies, including human saliva, human sputum, human stool, rex rabbits gut contents and water (Rawsthorne *et al*. 2009; Rogers *et al*. 2013; Li *et al*. 2017; Young *et al*. 2017; Fu *et al*. 2018; Marotz *et al*. 2018; Papanicolas *et al*. 2019). Recently, a study investigating the gut microbiota of Atlantic salmon (*Salmo salar*) using PMA showed that up to 9·1% of the sequencing reads came from nonviable bacterial cells (Dvergedal *et al*. 2020). However, this study only investigated the influence of PMA treatment on digesta samples, thus the application of PMA treatment on other fish tissues, such as mucosal samples with high levels of resident microbes, remains unknown.

Here, we characterized the intestinal bacterial communities in both gut contents and mucosal samples from farmed yellowtail kingfish (*Seriola lalandi*) using next generation 16S rRNA gene sequencing. We also assessed the microbial viability of yellowtail kingfish gut microbiota in both sample types using a modified PMA treatment protocol. The gastrointestinal tract is a physically perturbed and acidic environment, with food constantly moving through, epithelial cells being shed and microbial cells competing with each other for space and nutrients. As such, we hypothesized that numerous microbial cells (e.g. originating from the environment or food) become nonviable when exposed to this dynamic environment. Therefore, the intestinal content and gut mucosa would harbour complex bacterial communities comprising both viable (and likely active) and nonviable (not being able to survive in the gut environment) microbial cells.

## Materials and methods

### Sample collection

Five fish with a mean weight of 1·3 ± 0·1 kg and a mean length of 47 ± 1 cm were collected on the 18th of November 2019 from a single offshore seacage under the auspices of a commercial aquaculture enterprise of southern Australia according to industry best practice veterinary care. Fish originated from the same cohort and were fed the same proprietary diet prior to sampling. Fish were immediately transported on ice to a laboratory for dissection. Within 4 h post‐mortem, the body cavity of the fish was opened, and the digestive tract was extracted. The digesta was then collected by squeezing the gastrointestinal tract into a sterile 15 ml falcon tube and immediately placed on ice. Then, an incision was made along the length of the hindgut and midgut sections using a sterile scalpel blade to expose the inner mucosal surface. The exposed mucosa was then collected using a sterile glass microscope slide as described previously (Legrand *et al*. 2020c) and placed in a 50‐ml falcon tube. Gloves were used and changed between the collection of each sample type to avoid contamination.

For all digesta samples, 5 ml of digesta was placed into 15 ml falcon tubes containing 10 ml of phosphate buffered saline (Invitrogen^TM^ PBS, ThermoFisher Scientific, San Jose, CA, USA), and all samples were homogenized by vortexing. Samples were then centrifuged at 500 ***g*** for 3 min to remove debris using the Eppendorf 5810R centrifuge (Eppendorf, Hamburg, Germany). The supernatant was collected using a sterile pipette for each sample and transferred into a new 15 ml falcon tube. The clean samples were then centrifuged at 12 000 ***g*** for 8 min. The supernatant was subsequently collected and discarded. Finally, the cell pellet was resuspended in 2 ml of PBS, vortexed and split in two 1 ml aliquots in 1·5 ml centrifuge tubes. The samples were then placed back on ice until PMA treatment. For all mucosal samples, 3 ml of PBS was added to each 50 ml falcon tube while removing the glass microscope slide from the tube. Each tube was then vortexed vigorously and hand shaken until all the mucosa was well mixed with the PBS. Next, 1 ml of this solution was transferred into two 1·5 ml tubes (1 ml per tube) and placed on ice until PMA treatment.

### PMA treatment

Prior to PMA treatment, all samples were left at room temperature for 5 min. Then, half of the digesta and mucosal samples were placed back on ice and were used as nontreated (control) samples to investigate the influence of PMA on the resultant bacterial community composition. For the other half of the samples, 50 *µ*l of a solution containing 0·2 mmol l^−1^ of PMA (PMAxx^TM^, Biotium Inc, Hayward, CA, USA) was added in order to obtain a final concentration of 10 *µ*mol l^−1^ of PMA, as described previously (Marotz *et al*. 2018). Samples were then gently vortexed and incubated in the dark at room temperature for 5 min. Next, samples were laid horizontally on ice at <30 cm from a light source comprising two 500W halogen globes (Philips Plusline S 500W R7s) for a period of 25 min, with brief mixing of the samples every 5 min. Samples were then placed back on ice with the control samples. All samples were subsequently stored at −20°C until DNA extraction.

### DNA extraction, library preparation and sequencing

Prior to DNA extraction, all 20 samples (10 PMA‐treated and 10 controls from 5 digesta and 5 mucosal samples) were thawed at air temperature and mixed with light vortex. Then, 1 ml of each sample was used as input for DNA extraction using the MP Bio Fast DNA Spin Kit for faeces (MP Biomedicals, Solon, OH, USA) following the manufacturer's instructions. All DNA extracts were subsequently purified by ethanol precipitation using standard procedures and quantified using the NanoDrop 2000 spectrophotometer (ThermoFisher Scientific) and stored at −20°C.

Sample DNA extracts were sent to the Australian Genome Research Facility (AGRF, Melbourne, Australia) for Illumina NGS library preparation. The V3–V4 hypervariable region of the 16S rRNA gene was amplified from the purified DNA samples using the universal eubacterial primers 341F/806R (Takahashi *et al*. 2014). PCR products were then indexed using Nextera XT indexes, and libraries were normalized and pooled in equimolar concentrations for sequencing on the Miseq platform (Illumina, San Diego, CA, USA) using 300 bp paired‐end sequencing chemistry. Raw demultiplexed sequencing data with sample annotation were deposited in the NCBI SRA data repository under the BioProject ID PRJNA681418.

### Bioinformatics and statistical analysis

Sequencing of the 20 samples resulted in a total of 2 602 389 paired end reads (130 119 ± 34 150 per sample). Demultiplexed sequences were processed using QIIME2 (v.2019.10) (Bolyen *et al*. 2019). First of all, paired‐end reads were imported using the Casava 1·8 format. Then, forward reads were truncated to 297 bp and reverse reads to 223 bp to remove low‐quality sequences and denoised into amplicon sequence variants (ASVs) using the DADA2 plugin (Callahan *et al*. 2016). This resulted in a total of 2 134 733 merged reads retained for downstream analysis. Taxonomy was assigned to each ASV using the q2‐feature‐classifier against the Silva 132 99% OTUs (Operational Taxonomic Units) reference sequences resource (Quast *et al*. 2013). ASVs with <10 reads, as well as those which were unassigned or which represented mitochondria, chloroplast, eukaryote sequences, were removed from the dataset. Samples were rarefied to an even depth of 40 000 reads, resulting in a total of 1709 ASVs. Alpha rarefaction revealed sufficient sequencing coverage (Fig. S1). The plugin q2‐diversity was used to measure both alpha diversity metrics (Simpson diversity and observed ASVs) and beta diversity metrics (e.g. weighted and unweighted UniFrac). Functional profiles of the microbiome were predicted with Tax4Fun2 based on the KEGG database (Wemheuer *et al*. 2020). For univariate measures, statistical differences were assessed using the Wilcoxon rank‐sum test. For multivariate measures, the function permdisp was first used to check the assumption of homogeneous dispersion between groups. When the assumption was met, a permanova was conducted using the function Adonis (allowing for type III (partial) sums of squares, fixed effects of sum to 0 for mixed terms, and exact *P*‐values generated using unrestricted permutation of raw data) to measure for statistical differences between groups, accounting for both the treatment group (e.g. sample type or PMA treatment) and fish id (Anderson 2001). Due to the relatively low number of replicates between groups, differential abundance for each ASV was assessed using Deseq2 with Benjamini‐Hochberg false discovery rate method applied to correct the *P*‐values (Love *et al*. 2014; Weiss *et al*. 2017).

## Results

### Sample type influences the fish gut microbiota

**Figure 1:**
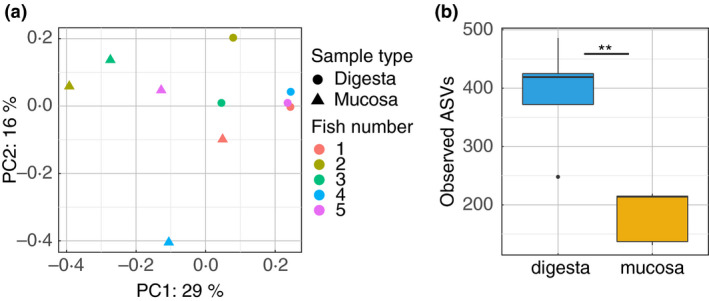
Impact of sample type on the global bacterial communities associated with the fish gut. (a) PCoA plot representing unweighted Unifrac distances comparing the change in bacterial communities found in digesta and mucosal samples for all five fish replicates used in this study. (b) Boxplot presenting the median and IQR of the number of observed amplicon sequence variants (ASVs) identified in digesta and mucosal samples. The levels of significant difference are denoted by *P ≤ 0·05, **P ≤ 0·01 and ***P ≤ 0·001, following the Wilcoxon rank‐sum test.

We first investigated the influence of sample type on the fish gut bacterial communities using the 10 control samples (five digesta and five mucosa samples). Using the unweighted Unifrac matrix, we found that the bacterial community composition was significantly different according to sample type (F.model = 2·26, *P* = 0·008) (Fig. 1a). This indicates that different microbial communities populate the mucosa of fish, compared to the digesta.

To further characterize this change in bacterial communities, we compared the alpha diversity in the two sample types. Simpson's diversity was higher in digesta than mucosal samples though not significant (*P* = 0·056, Fig. S2a). This was supported by significantly higher richness (observed Amplicon Sequence Variants ASVs, *P* = 0·008; Fig. 1b) and evenness (Pielou's evenness, *P* = 0·016; Fig. S2b) in the digesta samples compared to mucosal samples. This suggests that the fish mucosal samples contain fewer microbial species than digesta samples.

**Figure 2:**
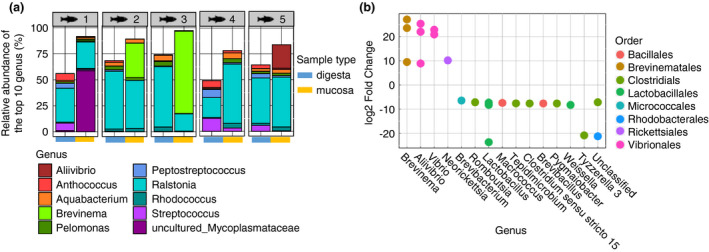
Impact of sample type on the taxonomical composition of the fish gut microbiota. (a) Stacked barplot presenting the relative abundance (%) of the top 10 most abundant bacterial genus found in the gut microbiota of all five replicates, comparing digesta and mucosal samples. (b) Dotplot showing significantly differentially abundant amplicon sequence variants (ASVs) between digesta and mucosal samples, as identified using Deseq2.

We also explored taxonomic differences between the two sample types (Fig. 2a). While *Ralstonia* was the most prevalent genus in both sample types, both sample types contained distinct bacterial taxa (Fig. 2a). Specifically, 23 differentially abundant ASVs were identified between these sample types (Table S1). *Brevinema*, *Aliivibrio* and *Vibrio* ASVs were significantly more prevalent in mucosa samples (Fig. 2b, Table S1). Clostridiales and Lactobacillales ASVs were more abundant in digesta samples (Fig. 2b, Table S1). Overall, these findings indicated that the type of biological sample examined can influence the microbiota signatures in the yellowtail kingfish gut microbiota.

The prediction of the functional profiles of all samples was generated using an average of 73 ± 22% of all sequences. However, in some samples, predictions were made using as low as 20% of the sequences (as observed in a mucosal sample, Table S2). In total, Tax4Fun2 was able to generate predictions for 356 KEGG pathways (Table S3). However, no significant differences in the functional profiles were found between digesta and mucosal samples.

### Sample treatment with PMA has an impact on the resulting microbiota in both digesta and mucosal samples

#### PMA treatment of digesta samples

**Figure 3:**
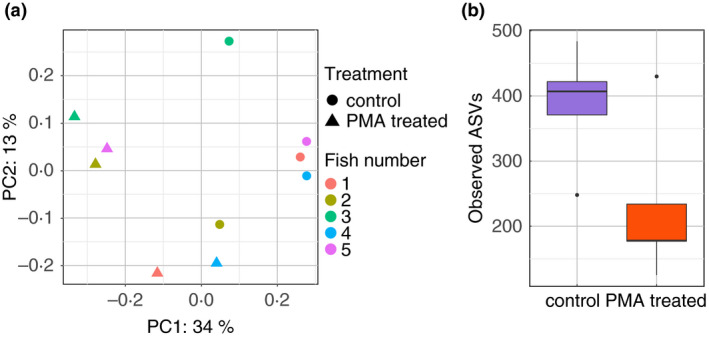
Impact of propidium monoazide (PMA) treatment on the global bacterial communities associated with the fish digesta. (a) PCoA plot representing unweighted Unifrac distances comparing the change in bacterial communities found in PMA‐treated and control samples for all five digesta replicates used in this study. (b) Boxplot presenting the median and IQR of the number of observed amplicon sequence variants (ASVs) identified in PMA‐treated and control digesta samples. The levels of significant difference are denoted by *P ≤ 0·05, **P ≤ 0·01 and ***P ≤ 0·001, following the Wilcoxon rank‐sum test.

Since both digesta and mucosal samples comprised distinct bacterial communities, we evaluated the influence of PMA treatment on these two sample types separately. Using the unweighted Unifrac distance matrix, PMA treatment had a significant effect on the global bacterial communities of digesta samples (F.model = 2·98, *P* = 0·013) (Fig. 3a).

PMA treatment also had a slight, yet nonsignificant, impact on the bacterial alpha diversity of digesta samples, as marked by a lower Simpson diversity in the PMA‐treated samples (*P* = 0·056, Fig. S3a). Untreated samples exhibited higher Pielou's evenness (*P* = 0·056, Fig. S3b) and higher numbers of observed ASVs (*P* = 0·095, Fig. 3b) although not significant. This indicates that several ASVs were detected from nonviable cells in digesta samples.

**Figure 4:**
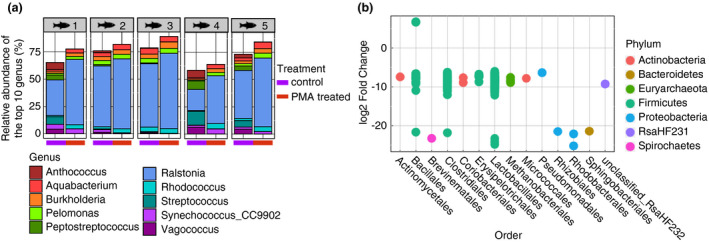
Impact of propidium monoazide (PMA) treatment on the taxonomical composition of the digesta‐associated microbiota. (a) Stacked barplot presenting the relative abundance (%) of the top 10 most abundant bacterial genus found in the digesta of all five fish replicates, comparing PMA‐treated and control samples. (b) Dotplot showing significantly differentially abundant amplicon sequence variants (ASVs) between PMA‐treated and control digesta samples, as identified using Deseq2.

The taxonomic composition of the samples post PMA treatment was changed (Fig. 4a). Typically, *Ralstonia* was more abundant in PMA‐treated digesta samples (Fig. 4a). Some taxa were not observed in PMA samples such as *Anthococcus*, *Streptococcus*, *Peptostreptococcus* and *Vagococcus* (Fig. 4a). In total, 153 AVS were found to be significantly differentially abundant between PMA‐treated and control digesta samples (Table S4). Among those, only two taxa were more prevalent in PMA‐treated samples and were *Brevibacillus* and *Staphylococcus* ASVs from the Bacillales order (Fig. 4b). Most of the ASVs found to be more abundant in control samples were associated with Bacillales, Clostridiales and Lactobacillales (Fig. 4b). Of particular note, 12 ASVs associated with *Lactobacillus*, 12 with *Enterococcus*, 5 with *Lactococcus*, 16 with *Streptococcus*, 8 with *Vagococcus* and 3 *Methanobrevibacter* were significantly less abundant in PMA‐treated samples. While the total relative abundance of Lactobacillales‐related ASVs in control samples was about 22%, the relative abundance of the same ASVs decreased to 0% in PMA‐treated samples (Fig. S4). No significant differences were observed in the functional profiles between PMA‐treated and nontreated digesta samples. Overall, this suggests that PMA treatment impacts composition, but not diversity, of gut digesta samples.

#### PMA treatment of mucosal samples

**Figure 5:**
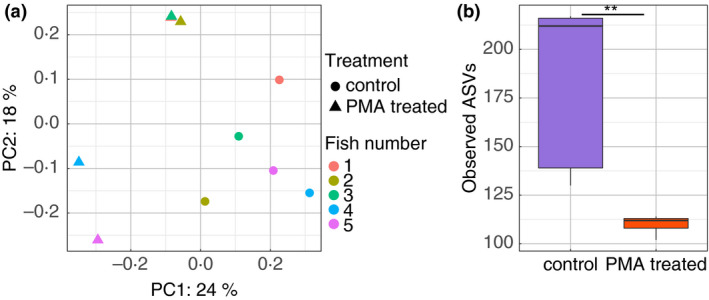
Impact of propidium monoazide (PMA) treatment on the global bacterial communities associated with the fish gut mucosa. (a) PCoA plot representing unweighted Unifrac distances comparing the change in bacterial communities found in PMA‐treated and control samples for all five mucosal replicates used in this study. (b) Boxplot presenting the median and IQR of the number of observed amplicon sequence variants (ASVs) identified in PMA‐treated and control mucosal samples. The levels of significant difference are denoted by *P ≤ 0·05, **P ≤ 0·01 and ***P ≤ 0·001, following the Wilcoxon rank‐sum test.

Next, we explored the impact of PMA treatment on the mucosal samples. Similar to the digesta samples, the bacterial community composition of mucosal samples were significantly different after PMA treatment when using the unweighted Unifrac distance matrix (F.model = 2·19, *P* = 0·002) (Fig. 5a). This indicates that PMA treatment significantly impacted the bacterial composition of gut mucosal samples.

PMA treatment was found to significantly decrease the number of ASVs identified in mucosal samples (observed ASVs, *P* = 0·008) (Fig. 5b). However, PMA treatment did not affect the Simpson's diversity (Simpson diversity, *P* = 1; Fig. S5a) or evenness (Pielou's evenness, *P* = 1; Fig. S5b) of the mucosal samples. This suggests that similarly to digesta samples, a number of ASVs detected in mucosal samples originated from nonviable cells.

**Figure 6:**
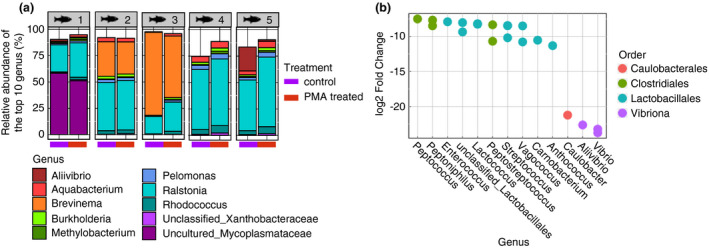
Impact of propidium monoazide (PMA) treatment on the taxonomical composition of the gut mucosa associated microbiota. (a) Stacked barplot presenting the relative abundance (%) of the top 10 most abundant bacterial genus found in the mucosa of all five fish replicates, comparing PMA‐treated and control samples. (b) Dotplot showing significantly differentially abundant amplicon sequence variants (ASVs) between PMA‐treated and control mucosal samples, as identified using Deseq2.

PMA treatment impacted the downstream taxonomical composition of the mucosal samples. Although the relative abundance of the most dominant taxa (e.g. *Ralstonia*, *Brevinema* and uncultured Mycoplasmataceae) remained similar between PMA‐treated and control samples, some ASVs were not detected in PMA‐treated samples (Fig. 6a). More specifically, 21 ASVs were significantly reduced in PMA‐treated samples (Fig. 6b, Table S5). Most of these ASVs were associated with Clostridiales, Lactobacillales and Vibrionales (Fig. 6b). Similarly to digesta samples, ASVs associated with *Enterococcus*, *Lactococcus*, *Streptococcus* and *Vagococcus* were lost after PMA treatment. Furthermore, the total relative abundance of Lactobacillales related ASVs decreased from 4% in control samples to 0% in PMA‐treated samples (Fig. S6), although these species were not as abundant as in digesta samples. No significant differences were observed in the functional profiles between PMA‐treated and nontreated mucosal samples. Overall, this suggests that PMA treatment significantly influenced the downstream microbial composition and diversity of gut mucosal samples.

## Discussion

Faecal (digesta) material is often used as a proxy in animal gut microbiome investigations due to it being a non‐invasive method in contrast to collecting the gut mucosa (Tang *et al*. 2020). However, there are major drawbacks when using this sample type such as incomplete separation between faecal bacteria and mucosal microbiota, homogenization of the sample and the effect of storage method (Tang *et al*. 2020). For instance, studies have shown that the mucus layer and intestinal lumen host distinct intestinal microbial niches with different biological roles in humans and other animals like dairy cattle and mice (Li *et al*. 2015; Mao *et al*. 2015; Ringel *et al*. 2015). In yellowtail kingfish, studies investigating the gut microbiota have used digesta, as well as a wide range of other sample types, including whole larvae, whole intestinal tract and intestinal mucosa (Wilkes Walburn *et al*. 2018; Horlick *et al*. 2020; Legrand *et al*. 2020c). In this study, we first investigated whether the microbial communities of digesta samples were different to those associated with mucosal samples. Based on both alpha and beta diversity indices, the two sample types exhibited distinct bacterial communities. We found a higher diversity and richness in digesta samples when compared to mucosa, a feature also identified in Atlantic salmon (Gajardo *et al*. 2016). However, this contrasts with previous results found in yellowtail kingfish, where a higher microbial diversity and richness were found in mucosal samples when compared to digesta samples (Horlick *et al*. 2020). Such variation within the same fish species can be explained with differences in environmental conditions (e.g. surrounding water, temperature), diet and number of replicates (Legrand *et al*. 2020b; Panteli *et al*. 2020). Taken together, this highlights the need to select the right sample type in relation to the research question when investigating fish gut microbiome. Typically, digesta is often collected when exploring transient (allochthonous) microbes that are influenced by environmental factors (e.g. diet or surrounding water) (Legrand *et al*. 2020b). In contrast, the mucosa contains more resident (autochthonous) microbes that are more influenced by the host and therefore more closely interact with the host mucosal surfaces (Ringo *et al*. 2016).

Another major limit of current fish microbiome studies is the inability to differentiate viable and nonviable microbial cells. There are several methods available to explore the bacterial viability in gut samples (e.g. plate counts, fluorescence approaches, staining of dead/viable cells), but these techniques are rarely used in current studies because of the expense of sequencing approaches (Hammes *et al*. 2010). While sequencing techniques focusing on RNA, proteins and metabolites (e.g. metatranscriptomics, metaproteomics and metabolomics) provide information on active microbial communities, they are not commonly used in studies exploring the fish microbiota due to their high cost and limited bacterial cells in specific sample type (e.g. mucosa) (Ghanbari *et al*. 2015; Legrand *et al*. 2020b). Instead, DNA‐based techniques (e.g. 16S rRNA gene and shotgun sequencing) are often used, but these methods cannot determine the viability and thus likely activity of microbial communities. This is of particular interest for studies that aim to assess the potential functional roles of the fish microbiome using predictive tools, such as PICRUSt or Tax4Fun, as sequencing data from total DNA will result in predicting the role of both active and non‐active microbial communities (Langille *et al*. 2013; Asshauer *et al*. 2015). As such, we investigated the use of PMA treatment in order to estimate the viability of the microbial communities found in digesta and mucosal samples.

In this study, we used a modified PMA treatment protocol and found that there were significant changes in bacterial communities between PMA‐treated and untreated digesta and mucosal samples. In both sample types, the composition of bacterial communities was significantly different after PMA treatment, as shown with the unweighted Unifrac matrix. In addition, we found a lower microbial richness and evenness in PMA‐treated samples, indicating that some microbes were not detected in PMA‐treated samples. More specifically, we found some bacterial lineages that were significantly reduced in PMA‐treated samples, which would imply that these microbes are not viable (and thus not active) in the fish gut microbiota. For instance, several ASVs associated with Bacillales, Clostridiales and Lactobacillales were significantly less abundant in digesta samples following PMA treatment. This trend was also observed in mucosal samples, where several Clostridiales and Lactobacillales were significantly less abundant in PMA‐treated samples. This is of particular interest to therapeutic treatments in aquaculture, as these lactic acid bacteria (LAB) are often considered as favourable micro‐organisms due to their beneficial roles in enhancing immune responses, disease resistance, digestive functions and mucosal tolerance (Ringo *et al*. 2018). Interestingly, several LAB‐associated genera that were depleted after PMA treatment (such as *Carnobacterium*, *Streptococcus*, *Enterococcus* and *Lactococcus*) are also known to contain potential pathogens (Ringo *et al*. 2018). As 16S rRNA gene sequencing is limited in its taxonomic resolution, further work should be implemented to better characterize the role of these important microbes found in the yellowtail kingfish gut microbiota.

While some bacterial lineages seem to be associated with nonviable cells, this study characterized the viability of the fish gut microbiota at only one point in time. Recently, it was revealed that time following feeding is an important driver of fish microbiota structure and functionality, as shown in clownfish (*Premnas biaculeatus*) and coral trout (*Plectropomus leopardus*) (Mekuchi *et al*. 2018; Parris *et al*. 2019). In this experiment, fish were collected in the morning, and their gastrointestinal tract contained leftover food from the previous day. As a result, bacterial viability could have been different if sampling occurred at a different time following feeding. Thus, the influence of feeding retention on bacterial viability requires further elucidation. In addition, it is unknown whether the nonviable bacteria detected in this study came from the environment/food or were already established in the fish gastrointestinal tract. In Atlantic salmon, it was demonstrated that diet and seawater derived bacteria were found in the fish hindgut (Zarkasi *et al*. 2016). It is therefore possible that the nonviable bacteria found in the yellowtail kingfish gut microbiota originate from feed or the surrounding environment. Despite this, LAB are typically occurring in the fish gut microbiota, as observed in numerous fish species (Ringo *et al*. 2018; Wang *et al*. 2018). In this study, LAB was more abundant in digesta samples than mucosal samples. This suggests that LAB are prevalent in the fish allochtonous bacterial communities and therefore not in close interactions with the host, in contrast to the autochtonous micro‐organisms. Considering that the gastrointestinal tract is a complex environment where microbes are constantly under pressure (e.g. host‐microbe and microbe–microbe interactions) (Perez *et al*. 2010; Legrand *et al*. 2020b), it remains unclear if these non‐viable cells were previously viable before sampling. In addition, no differences in the predicted functional profiles were found between sample type and PMA treatment. This result could be explained by the low number of replicates used in this study (5 per treatment group) and poor level of prediction in some samples, as no reference genome for some of the bacteria found in the gut microbiota of yellowtail kingfish are available in the database used to generate the predictions. Therefore, further studies including the collection of samples at different time points, as well as exploring the gene expression and metabolite profile of these communities, are required to better elucidate the role of these micro‐organisms within the fish gastrointestinal tract.

Overall, this study highlights important caveats found in fish microbiota related studies. Here, we showed that the digesta and gut mucosa contain distinct bacterial communities. As such, care should be taken when selecting sample type to investigate the fish gut microbiota. While collecting digesta has the advantage of being a non‐invasive method, collecting the gut mucosa seems more appropriate if the overall aim of the study was to explore the micro‐organisms that are in closer interaction with the host. In addition, the microbial activity of the fish gut microbiota is likely to have an influence on the resultant role of these communities in disease resistance and nutrient digestibility, and ultimately fish health and performance. As a result, characterizing the active microbial communities found in the fish microbiota is paramount. In this regard, PMA treatment can be a very useful, cost effective tool. This simple, rapid and cost‐effective method can easily be applied to better characterize and understand the contribution of dominant fish gut microbiota constituents.

## Authors' contributions

T.P.R.A.L. conceptualized the study, collected samples, performed laboratory work, analysed the data and wrote the manuscript. A.P.A.O. wrote the manuscript and supervised the study. M.L.W. contributed to data interpretation and edited the manuscript. J.W.W. provided reagents, edited the manuscript and supervised the study. L.S.W. edited the manuscript and supervised the study.

## Conflict of Interest

No conflict of interest declared.

## Supporting information


**Figure S1** Rarefaction plot of all samples analysed in this study.
**Figure S2** Boxplot presenting the median and IQR of (a) Simpson's diversity and (b) Pielou's evenness in digesta and mucosal samples. The levels of significant difference are denoted by *P ≤ 0·05, **P ≤ 0·01 and ***P ≤ 0·001, following the Wilcoxon rank‐sum test.
**Figure S3** Boxplot presenting the median and IQR of (a) Simpson's diversity and (b) Pielou's evenness in PMA‐treated and control digesta samples. The levels of significant difference are denoted by *P ≤ 0·05, **P ≤ 0·01 and ***P ≤ 0·001, following the Wilcoxon rank‐sum test.
**Figure S4** Boxplot presenting the median and IQR of the relative abundances of the summed Lactobacillales associated ASVs found in PMA‐treated and control digesta samples. The levels of significant difference are denoted by *P ≤ 0·05, **P ≤ 0·01 and ***P ≤ 0·001, following the Wilcoxon rank‐sum test.
**Figure S5** Boxplot presenting the median and IQR of (a) Simpson's diversity and (b) Pielou's evenness in PMA‐treated and control mucosal samples. The levels of significant difference are denoted by *P ≤ 0·05, **P ≤ 0·01 and ***P ≤ 0·001, following the Wilcoxon rank‐sum test.
**Figure S6** Boxplot presenting the median and IQR of the relative abundances of the summed Lactobacillales associated ASVs found in PMA‐treated and control mucosal samples. The levels of significant difference are denoted by *P ≤ 0·05, **P ≤ 0·01 and ***P ≤ 0·001, following the Wilcoxon rank‐sum test.

**Table S1** Differentially abundant ASVs found in digesta and mucosal samples.
**Table S2** Amount of sequences used to generate the prediction of microbial functions using Tax4Fun2.
**Table S3** Prediction of the functional profiles of all samples used in this study.
**Table S4** Differentially abundant ASVs found in PMA‐treated and control digesta samples.
**Table S5** Differentially abundant ASVs found in PMA‐treated and control mucosal samples.
