# Supplementary material for: Dead or alive: microbial viability treatment reveals both active and inactive bacterial constituents in the fish gut microbiota
Source: J Appl Microbiol. 2021 May 4;131(5):2528–38. doi: 10.1111/jam.15113 (PMC8596808; doi:10.1111/jam.15113)
Supplement: Supplementary file 1 — Figure S1 Rarefaction plot of all samples analysed in this study. Figure S2 Boxplot presenting the median and IQR of (a) Simpson's diversity and (b) Pielou's evenness in digesta and mucosal samples. The levels of significant difference are denoted by *P ≤ 0·05, **P ≤ 0·01 and ***P ≤ 0·001, following the Wilcoxon rank‐sum test. Figure S3 Boxplot presenting the median and IQR of (a) Simpson's diversity and (b) Pielou's evenness in PMA‐treated and control digesta samples. The levels of significant difference are denoted by *P ≤ 0·05, **P ≤ 0·01 and ***P ≤ 0·001, following the Wilcoxon rank‐sum test. Figure S4 Boxplot presenting the median and IQR of the relative abundances of the summed Lactobacillales associated ASVs found in PMA‐treated and control digesta samples. The levels of significant difference are denoted by *P ≤ 0·05, **P ≤ 0·01 and ***P ≤ 0·001, following the Wilcoxon rank‐sum test. Figure S5 Boxplot presenting the median and IQR of (a) Simpson's diversity and (b) Pielou's evenness in PMA‐treated and control mucosal samples. The levels of significant difference are denoted by *P ≤ 0·05, **P ≤ 0·01 and ***P ≤ 0·001, following the Wilcoxon rank‐sum test. Figure S6 Boxplot presenting the median and IQR of the relative abundances of the summed Lactobacillales associated ASVs found in PMA‐treated and control mucosal samples. The levels of significant difference are denoted by *P ≤ 0·05, **P ≤ 0·01 and ***P ≤ 0·001, following the Wilcoxon rank‐sum test. [file JAM-131-2528-s002.pptx]

## Slide 1
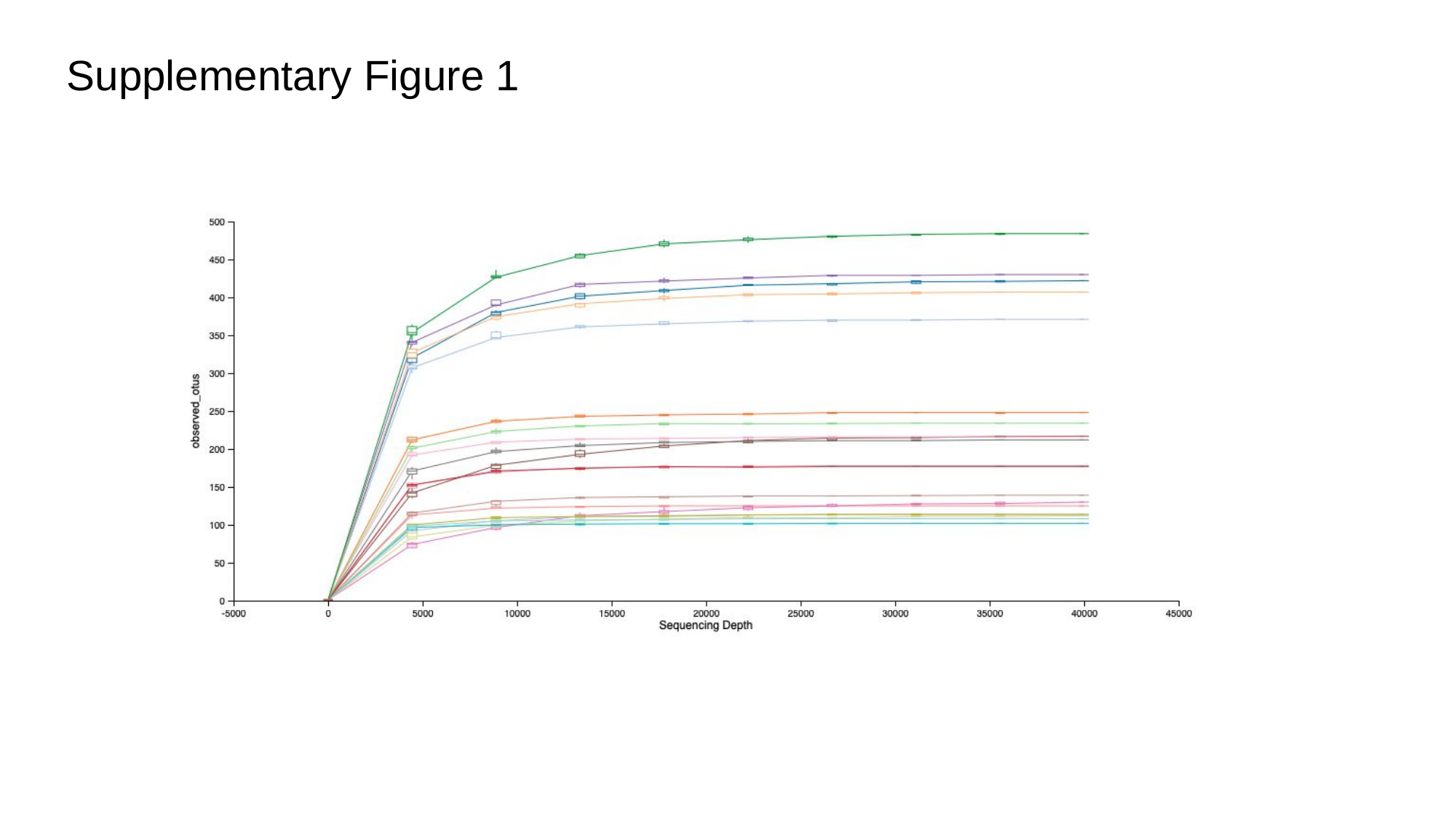

Supplementary Figure 1

## Slide 2
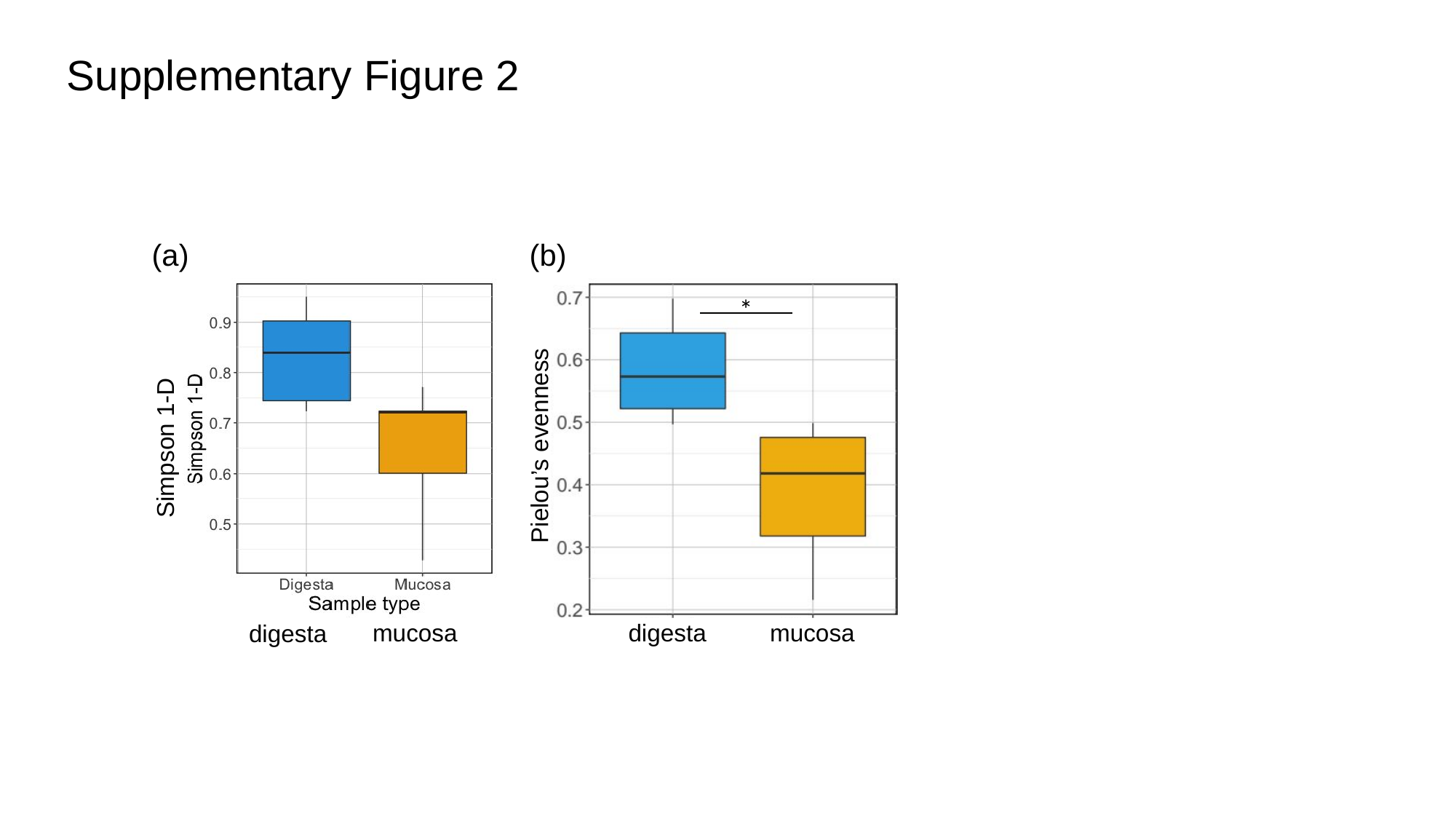

Supplementary Figure 2
(a)
(b)
**
*
Pielou’s evenness
Simpson 1-D
mucosa
digesta
mucosa
digesta

## Slide 3
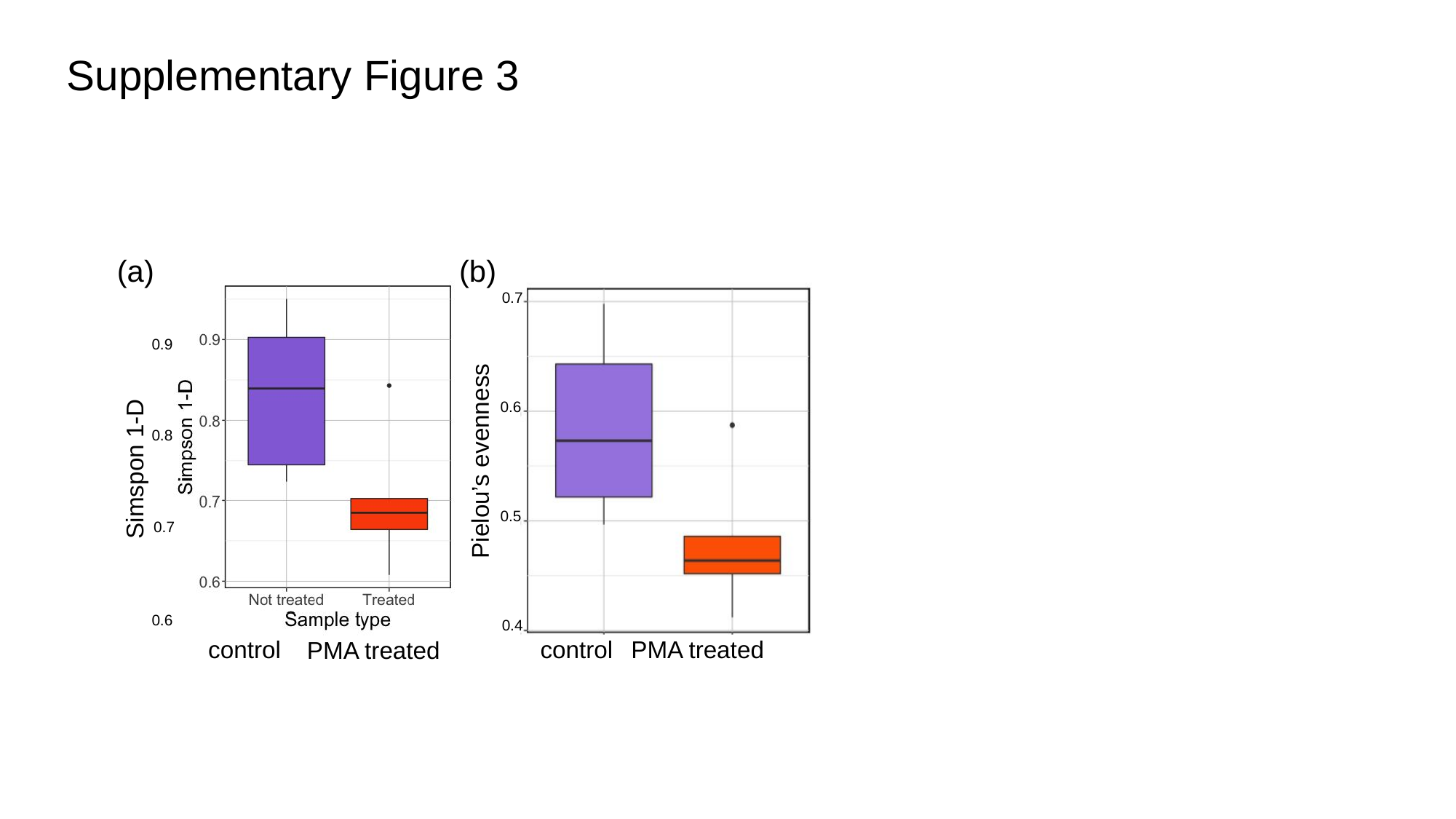

Supplementary Figure 3
(a)
(b)
0.7
0.6
Pielou’s evenness
Simspon 1-D
0.5
0.4
control
PMA treated
control
PMA treated
0.9
0.8
0.7
0.6

## Slide 4
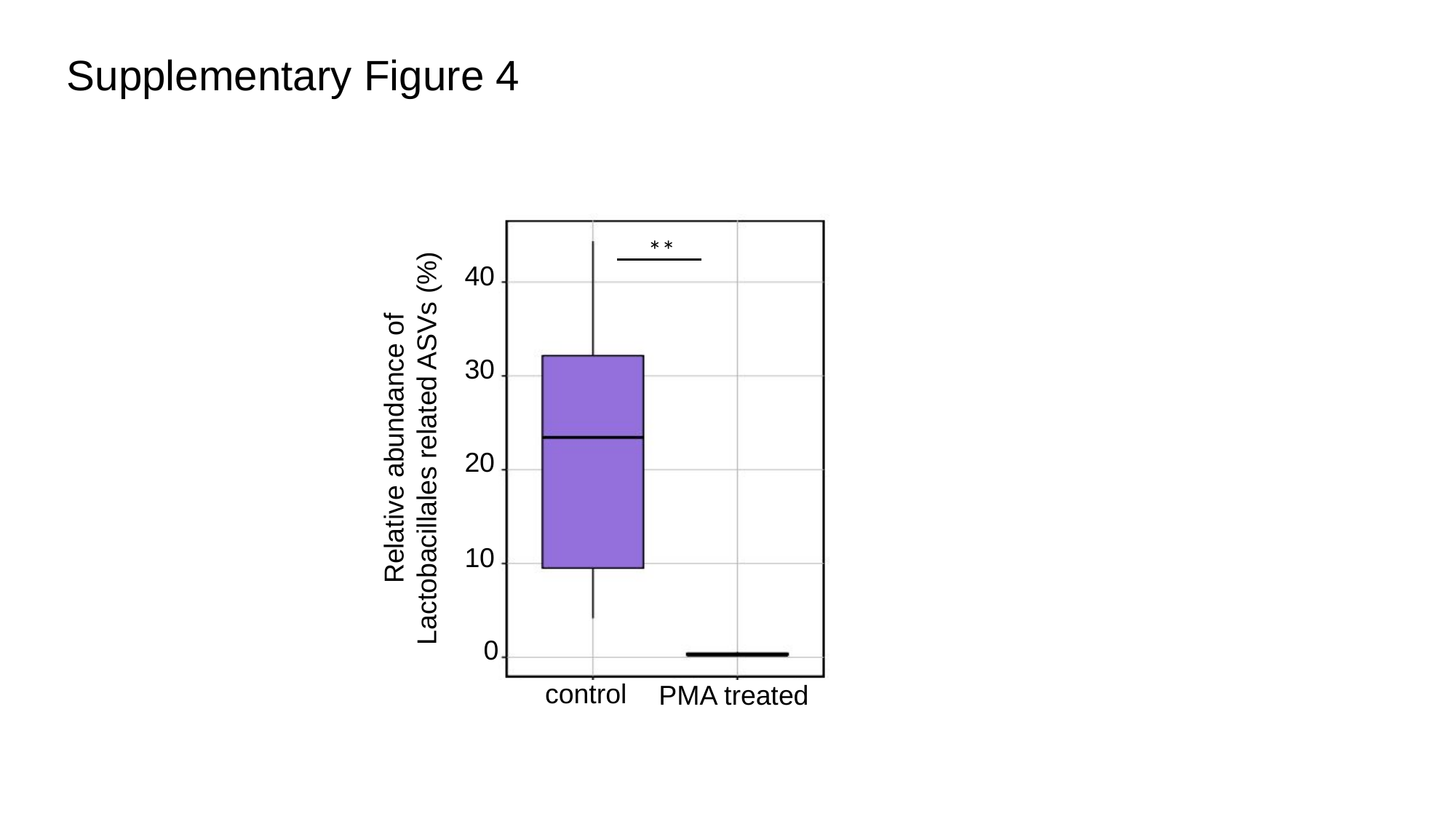

Supplementary Figure 4
**
40
30
Relative abundance of Lactobacillales related ASVs (%)
20
10
0
control
PMA treated

## Slide 5
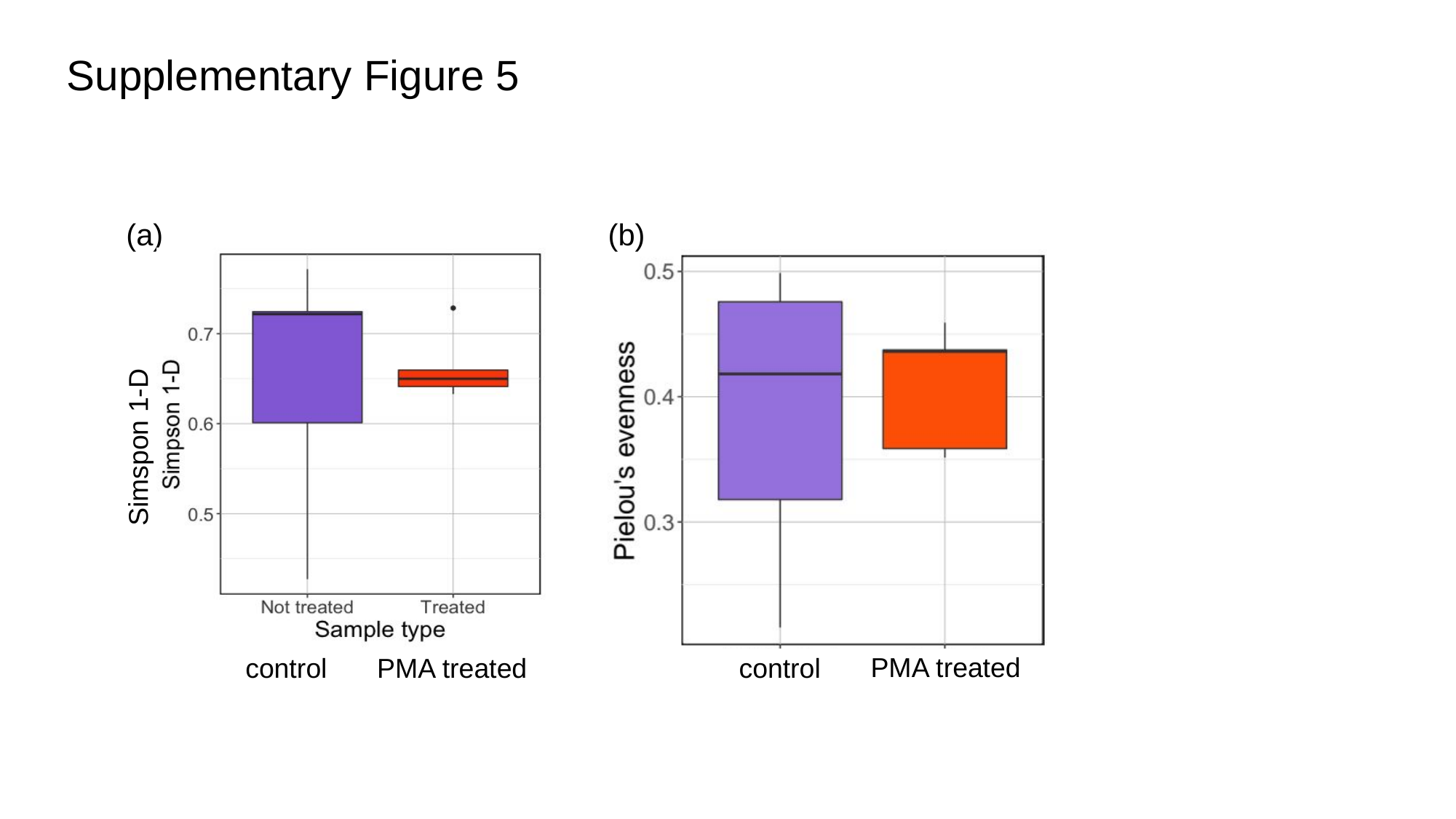

Supplementary Figure 5
(a)
(b)
PMA treated
PMA treated
control
control
Simspon 1-D

## Slide 6
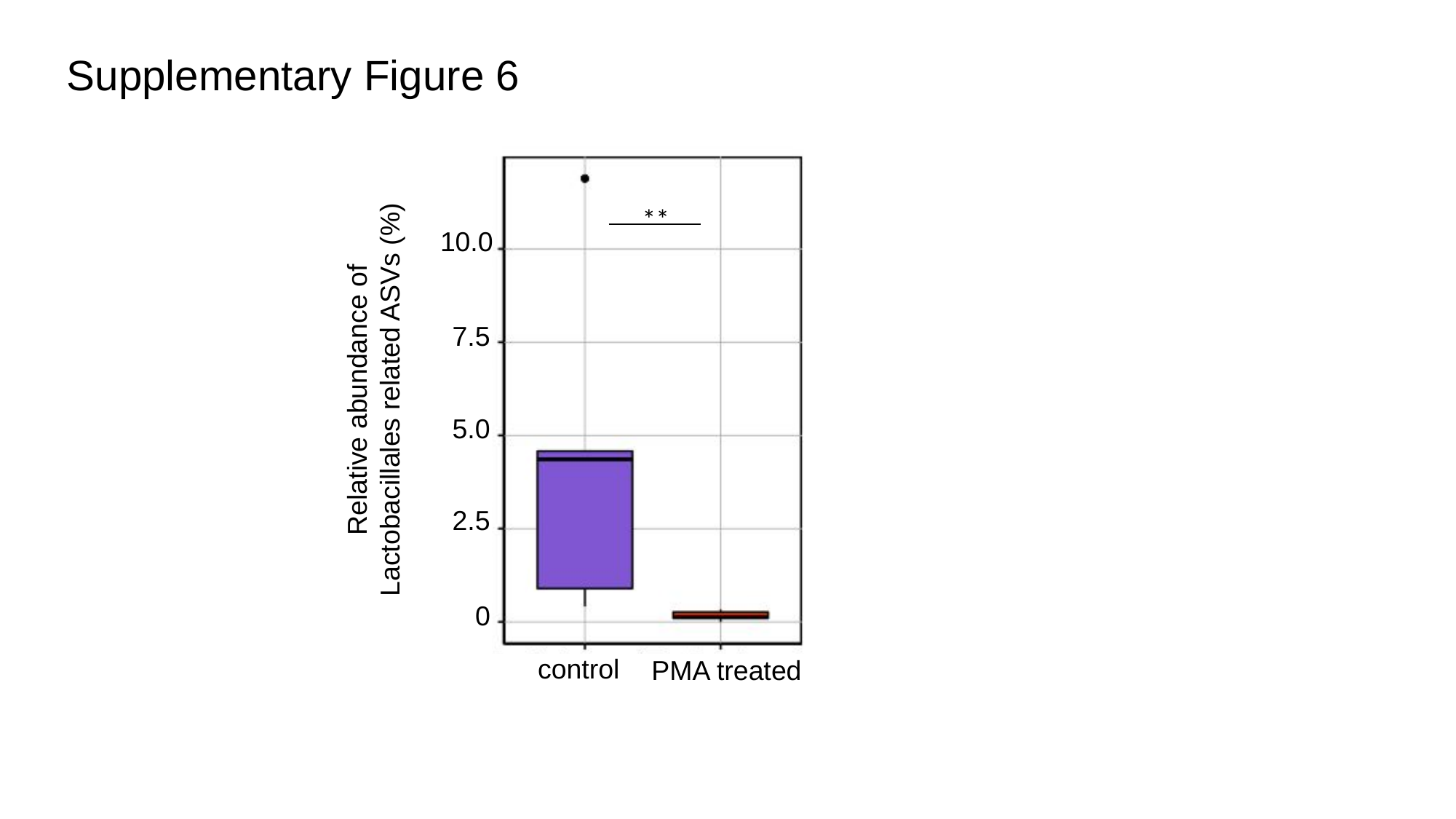

Supplementary Figure 6
**
10.0
7.5
Relative abundance of Lactobacillales related ASVs (%)
5.0
2.5
0
control
PMA treated
